# A retrospective study on development and internal validation of cardiovascular disease risk prediction model for patients with chronic kidney disease stage 3–5 within 5 years

**DOI:** 10.7717/peerj.21312

**Published:** 2026-06-05

**Authors:** Huixia Liu, Jing Xiong

**Affiliations:** Department of Nephrology, Union Hospital, Tongji Medical College, Huazhong University of Science and Technology, Wuhan, China

**Keywords:** Cardiovascular disease, Chronic kidney disease, Prediction model, Traditional risk factors, Non-traditional risk factors

## Abstract

**Background:**

Cardiovascular disease (CVD) is the leading cause of mortality in chronic kidney disease (CKD) patients. Traditional CVD risk factors exhibit diminished predictive utility in advanced CKD, necessitating integration of non-traditional biomarkers. Previous prediction models based only on traditional CVD risk show limitations and inaccuracies. This study aimed to develop and validate a 5-year CVD risk prediction model combining clinical, laboratory, and imaging parameters for CKD stages 3–5 patients.

**Methods:**

Three hundred and one patients with CKD stage 3–5 were recruited from January 2010 to January 2022 and followed up until July 2022. Least Absolute Shrinkage and Selection Operator (LASSO) regression and multivariable logistic regression were used to identify baseline predictors for model development including clinical data, medication history and laboratory parameters, regression modeling was performed using logistic regression and internally validated using tenfold cross-validation. Discrimination and calibration of resulting prediction models were assessed using c-statistic and *P*-value of the Hosmer-Lemeshow test. Decision curve analysis was performed to assess clinical effectiveness.

**Results:**

During follow-up, 169 (56.1%) experienced a first CVD event within 5 years. The median time of occurrence was 10 months. From 29 candidate variables, 11 independent predictors were identified. Through nested model comparisons, we demonstrated that adding inflammatory marker C-reactive protein (CRP) and echocardiographic marker interventricular septum thickness (IVS) markers to traditional risk factors progressively improved predictive performance. The full model—integrating clinical, inflammatory, and imaging parameters—achieved the highest discrimination (area under the curve (AUC), 0.845, 95% confidence interval (CI), [0.802–0.888]) and best fit (Akaike information criterion (AIC), 311.531), with excellent calibration (Hosmer-Lemeshow *P* = 0.332).

**Conclusions:**

This study established and validated a clinical risk prediction model based on readily available variables in clinical practice, aiming to predict the risk of CVD events in patients with CKD stages 3–5 over a 5-year period.

## Introduction

Chronic kidney disease (CKD) is currently considered to be one of the most common disorders globally, resulting in significant elevation of morbidity, mortality and health care costs (Saran et al., 2020). The prevalence of CKD in the global general population has reached 14.3% (Ene-Iordache et al., 2016). In China, the prevalence of CKD among adults is 10.8%. Accordingly, more than 160 million CKD patients are present in the adult Chinese population (Liyanage et al., 2022). Statistically, by the end of 2021, the number of patients with kidney failure requiring hemodialysis has exceeded 700,000 in China. Patients with CKD have an increased risk of progression to kidney failure requiring kidney replacement therapy (KRT), cardiovascular disease (CVD) events and death, with higher risk at higher CKD stages (Evans et al., 2018; Eckardt et al., 2018). CVD events are a major cause of morbidity and mortality in patients with all forms of CKD (Go et al., 2004). Current studies suggest that CKD is an independent risk factor for CVD events and is thought to be related to an increased risk of CVD events and higher all-cause mortality, and approximately half of CKD patients die from CVD events before progressing to kidney failure (Matsushita et al., 2010, 2015). Early detection and intervention in patients with CKD represents the best opportunity to improve outcomes. Normative management of patients with CKD could therefore delay the progression of CKD and reduce the risk of CVD events.

There have been several previous studies on developing and validating prediction models for predicting the CVD events. For example, the Framingham predictive instrument incorporates traditional risk factors affecting CVD events, including gender, age, blood pressure, cholesterol, history of diabetes mellitus and smoking, to predict the risk of CVD events and provide clinicians with a convenient assessment method. But whether it holds true in the CKD population remains unclear (Wilson et al., 1998; Wilson, Castelli & Kannel, 1987; D’Agostino et al., 2001, 2008). In 2011, Tangri et al. (2011) developed risk prediction models for kidney failure in patients with CKD stages 3–5 through two Canadian cohorts, and this kidney failure equation was validated in 31 global cohorts (Tangri et al., 2016). Currently, in 2018, Grams et al. (2018) developed a prediction model using data of 264,296 individuals in 30 countries participating in the international Chronic Kidney Disease Prognosis Consortium to predict non-fatal CVD events with estimated glomerular filtration rate (eGFR) under 30 ml/min/1.73 m^2^. In 2021, You et al. (2021) developed an economic nomogram for predicting cardiovascular risk in long-term hemodialysis patients that is of potential value for clinical application. More recently, Bundy et al. (2022) developed and validated a 10-year atherosclerotic cardiovascular disease risk prediction model specifically for CKD patients using the Chronic Renal Insufficiency Cohort study, incorporating both traditional risk factors and novel biomarkers such as high-sensitivity C-reactive protein and cardiac troponin. Likewise, Zelnick et al. (2021) employed machine learning approaches to predict incident atrial fibrillation in the same CKD cohort, demonstrating the incremental value of cardiac-specific biomarkers.

However, the existing models have certain limitations. First, some models are suitable for clinical assessment of the risk of CVD events in the general population and are not applicable to patients with CKD stages 3–5. Second, there are models that assess the absolute risk of kidney failure in patients with CKD that do not take the risk of CVD events into account in this population. In addition, the variables included in these models are often traditional risk factors that influence CVD events, such as gender, age, blood pressure, race, lipids, history of diabetes, smoking, and alcohol consumption; When non-traditional risk factors associated with CKD were included, such as serum creatinine (Scr), blood urea nitrogen (BUN), 24-h urine protein, electrolytes, Estimated Glomerular Filtration Rate (eGFR), hemoglobin (Hb), and inflammatory markers, the risk of incident CVD was more pronounced. Therefore, it is necessary to incorporate not only traditional risk factors related to the heart disease but also some non-traditional risk factors related to kidney disease when predicting the risk of CVD events in patients with CKD.

This study aimed to develop and validate a risk prediction model to explore the risk of CVD within 5 years in patients with CKD stages 3–5, combining traditional risk factors and non-traditional risk factors associated with CKD, which could help clinical practice in the standard management of CKD patients and reduce patients’ risk of developing adverse outcome events.

## Materials and Methods

### Ethical approval

This study was reviewed and approved by the Ethics Committee of Union Hospital, Tongji Medical College, Huazhong University of Science and Technology; Approval No. [2023] (Lunshenzi 0060). Data analysis adhered strictly to the approved ethical protocol. The need for informed consent was waived by the aforementioned ethics committee. Portions of this text were previously published as part of a preprint (https://doi.org/10.21203/rs.3.rs-4625793/v1).

### Study population

This retrospective cohort study utilized a tenfold cross-validated risk prediction model. The study cohort was derived from all eligible patients with stages 3–5 CKD who were regularly followed at Union Hospital, Tongji Medical College, Huazhong University of Science and Technology, a tertiary hospital in Wuhan, China. Time zero was defined as the date of the earliest documented diagnosis of CKD stage 3–5 in the patient’s medical record. Baseline clinical and laboratory data were collected from records within a window of 3 months before to 3 months after this index date. If multiple measurements were available within this period, the value closest to the index date was used. Patient recruitment spanned from January 2010 to January 2022, with a final follow-up in July 2022. In this study, regular follow-up was defined as the patient having at least one complete medical record (including medical history, physical examination, and necessary laboratory tests) in the nephrology outpatient clinic or inpatient department of our hospital every 6 months following a diagnosis of CKD stages 3–5. CKD diagnosis was established based on the 2012 KDIGO clinical practice guidelines (Stevens & Levin, 2013). The analysis included all enrolled subjects for model development, with internal validation performed *via* tenfold cross-validation.

**Figure 1:**
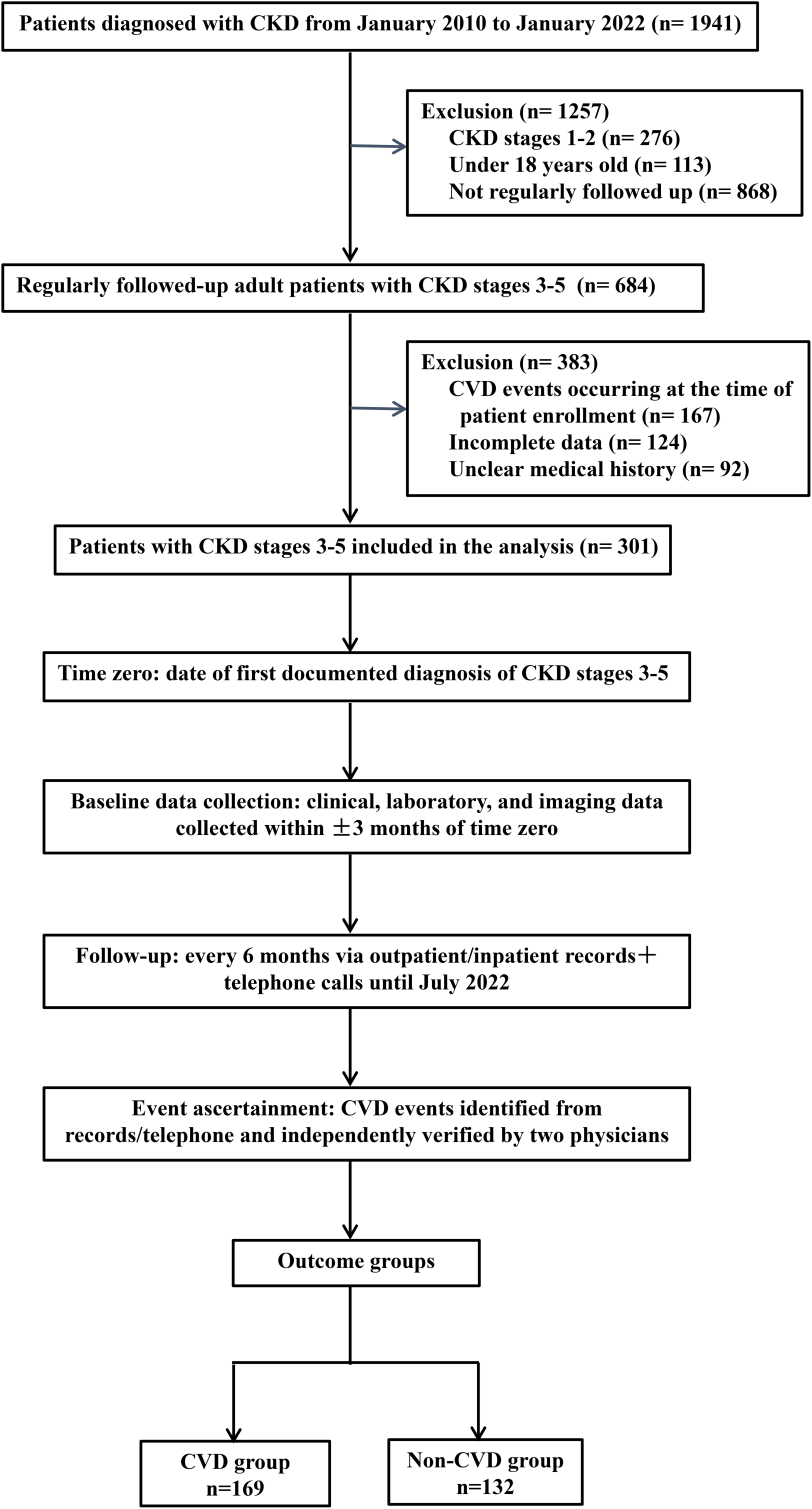
Inclusion and exclusion flowchart.

Eligibility required a diagnosis of CKD stages 3–5 at our institution, an age of 18 years or above, and adherence to regular follow-up protocols. Potential participants were excluded if they presented with prevalent CVD events at enrollment or had incomplete medical records or an unclear history. Patients receiving maintenance hemodialysis or peritoneal dialysis were not excluded from the study. A detailed flowchart of participant selection is provided in Fig. 1.

### Outcomes

The primary outcome events were fatal and non-fatal CVD events. In this study, CVD events were defined as acute myocardial infarction (I21–I22), unstable angina (I20.0), heart failure (I50, I11.0, I13.0, I13.2), ischemic stroke (I63, I64), hemorrhagic stroke (I60-I62), and cardiogenic shock (R57.0). Patients were followed up based on whether they had a CVD event within 5 years of diagnosis of CKD stages 3–5 as the endpoint, and endpoints were obtained from patient inpatient data, telephone follow-up visits on outpatient data. All potential outcome events were identified from inpatient records, outpatient records, and telephone follow-up visits. Each event was independently verified by two physicians based on the standardized diagnostic criteria listed above. Disagreements were resolved through consensus or consultation with a third senior cardiologist. All included patients were followed from the time of CKD stages 3–5 diagnosis until the earliest of the following: (a) the first occurrence of a cardiovascular disease event, (b) death from any cause, or (c) the last clinical visit before the administrative censoring date of June 30, 2022.

### Data collection

In this study, we defined the time zero as the point when patients were admitted and diagnosed with CKD stages 3–5. The index date (time zero) was defined as the date of the earliest documented diagnosis of CKD stage 3–5 in the patient’s medical record. Baseline clinical and laboratory data for model predictors were collected from records within a window of 3 months before to 3 months after this index date. If multiple measurements were available within this period, the value closest to the index date was used.

In the model construction, all data for the predictive variables were collected prior to time zero to ensure the accuracy and timeliness of the predictions. The selection of candidate predictive factors was primarily determined through a review of the literature, followed by the use of lasso regression and multivariable logistic regression analysis to screen the predictive factors. Ultimately, this study selected 11 predictive factors for model construction. Predictive factors for this study included the following clinical data: age, gender, body mass index (BMI), history of diabetes mellitus (DM) and hypertension, history of smoking and alcohol consumption, systolic blood pressure (SBP) and diastolic blood pressure (DBP). Diabetes mellitus was defined as type 2 diabetes based on clinical diagnosis. Patients with type 1 diabetes were not excluded but accounted for <5% of the cohort. Medication history: β receptor blockers, antiplatelet agents, renin angiotensin aldosterone system (RAAS) inhibitors, and lipid-lowering drugs. Laboratory parameters: Hb levels, platelet (PLT) levels, albumin (ALB) levels, BUN levels, Scr levels, uric acid (UA) levels, total cholesterol (TC) level, triglyceride (TG) levels, low-density lipoprotein-cholesterol (LDL-C) levels, blood calcium and phosphorus, 1/eGFR (per 0.01 unit increase), 24-h urine protein, parathyroid hormone (PTH), inflammatory indicators include C-reactive protein (CRP). Imaging metrics: cardiac interventricular septum (IVS) thickness.

To ensure the robustness of the prediction model, patients lacking essential baseline data required for model construction—specifically cardiac imaging (IVS thickness) and key laboratory tests (*e.g*., CRP, PTH) that necessitate collection within a defined time window-were excluded during the screening phase. This decision was based on the fact that such omissions typically represent non-random, systematic missingness (*e.g*., the test was not performed), which cannot be reliably imputed. For the final analytical cohort, missing values did not exceed 30% for all variables and were considered random. These were handled using multiple imputation as described in the Statistical Analysis section.

### Measurement methods

Measurement of key predictive variables followed standard clinical operating procedures. Office blood pressure was measured by trained nurses using a calibrated electronic sphygmomanometer. After the patient had rested in a seated position for 5 min, two consecutive measurements were taken on the right upper arm, and the average value was recorded. Cardiac echocardiography was performed by experienced sonographers using an ultrasound system. IVS thickness was measured at end-diastole in the parasternal long-axis view, obtained by two-dimensionally guided M-mode or direct two-dimensional measurement, in accordance with the guidelines of the American Society of Echocardiography.

### Statistical analysis

All statistical analyses were performed using R software (version 4.1.3; R Foundation for Statistical Computing, Vienna, Austria) (Liao et al., 2014). A two-sided *P*-value < 0.05 was considered statistically significant throughout the analysis.

### Data imputation and descriptive

A minimal amount of missing data (all variables had <30% missingness in the final cohort) was handled using multiple imputation *via* the mice package (five imputations, predictive mean matching method). The missing rates for the key variables were 11.3% for PTH, 19.6% for CRP, and 29.2% for IVS. Missingness was assumed to be missing at random (MAR), as the probability of missing data was unrelated to the outcome or other observed covariates after conditioning on available data. Due to the low overall missingness and the stability of imputation results across the five imputed datasets, no additional sensitivity analyses were performed. Pooled results from the imputed datasets were used for subsequent analyses. Categorical variables are presented as numbers (percentages) and compared using the chi-square test. Continuous variables are presented as mean ± standard deviation or median (interquartile range) based on their distribution and compared using the Student’s t-test or Mann-Whitney U test, as appropriate.

### Predictor selection and model development

To mitigate multicollinearity and select predictors, least absolute shrinkage and selection operator (LASSO) regression was first applied to all candidate variables. Variables with non-zero coefficients in the LASSO analysis were then entered into a multivariable logistic regression model. A backward stepwise selection procedure (with a significance level of *P* < 0.05 for retention) was used to construct the final model for predicting the 5-year risk of CVD events. This study employed a multivariable logistic regression model to estimate the cumulative risk probability of CVD events within 5 years following a diagnosis of CKD stages 3–5. We recognize that due to variations in patient enrollment times and follow-up durations, standard survival analysis models hold a theoretical advantage in utilizing individual follow-up time information. However, the primary aim of this study was to provide a straightforward 5-year fixed-time-point risk prediction tool for clinical use. The logistic regression model is widely applied in this context, and its results (odds ratio, OR) are more intuitive for clinicians to interpret.

### Model performance and validation

The predictive performance of the model was assessed in terms of discrimination, calibration, and clinical utility, including metrics such as the area under the ROC curve (AUC) and the Bayesian Information Criterion (BIC). Discrimination, referring to the model’s ability to distinguish between patients who did and did not experience a CVD event within 5 years, was evaluated using the area under the receiver operating characteristic curve (AUC) and the concordance statistic (C-statistic). Calibration, which reflects the agreement between predicted probabilities and observed outcomes, was assessed *via* calibration plots, the calibration slope and intercept, and the Hosmer-Lemeshow goodness-of-fit test. The assessment of explained variation, though part of a complete validation framework, was not the primary focus of this study, which prioritized the clinically interpretable measures of discrimination and calibration.

### Internal validation

To account for overoptimism (overfitting), internal validation was conducted. The preferred method for this involves bootstrap resampling, where the entire modeling process (including imputation, variable selection, and model fitting) is repeated in numerous bootstrap samples to calculate optimism-corrected performance estimates. In this study, we employed 10-fold cross-validation to provide a robust estimate of the model’s predictive performance on unseen data and to assess the stability of the selected predictors. The average performance metrics across all folds are reported (Jung & Hu, 2015).

### Model presentation

Based on the results of statistical analysis in this study, a nomogram was constructed to predict the risk of CVD events within 5 years in patients with CKD stages 3–5. Nomograms are used as follows: each variable value is assigned a score by drawing a line from its corresponding variable value into the “point” line. Then, the scores of each variable were summed, and the corresponding values were found on the “total point” axis, transforming the complex equation into an intuitive graph, facilitating its practical value in the clinic.

## Results

### Baseline characteristics

A total of 301 patients with clinical diagnosis of CKD stages 3–5 were included in this study, including 167 males and 134 females. Among them, 59 (19.6%) were classified as CKD stage 3, 85 (28.2%) as stage 4, and 157 (52.2%) as stage 5. In the CVD group (*n* = 169), the distribution was: stage 3, 26 (15.4%); stage 4, 55 (32.5%); stage 5, 88 (52.1%). In the non-CVD group (*n* = 132), the distribution was: stage 3, 33 (25.0%); stage 4, 30 (22.7%); stage 5, 69 (52.3%). At baseline, 114 patients (37.8%) were receiving maintenance dialysis (hemodialysis or peritoneal dialysis). During the follow-up period until August 2022, there were 169 outcome events (56.1%), of which 1 (0.06%) developed unstable angina, 24 (73.4%) heart failure, 11 (6.5%) myocardial infarction, 21 (12.4%) coronary heart disease, 6 (3.6%) cerebral infarction, 3 (1.8%) cerebral hemorrhage, and 3 (1.8%) cardiogenic shock. Groups were stratified based on whether CVD events occurred within 5 years in patients with CKD stages 3–5, with group 1 being patients with CKD stages 3–5 without CVD events and group 2 being patients with CKD stages 3–5 with CVD events. The baseline characteristics of the three hundred and one CKD patient subgroups are presented in Table 1. Compared with patients who did not experience a CVD event, those who experienced a CVD event had lower DBP and Hb levels, higher LDL, Scr, CRP, and IVS levels, and less use of antiplatelet agents, RAAS blockers, and lipid-lowering medications.

**Table 1:** General characteristics of 301 patient s between CVD and non-CVD group.

	Variables	Total (*n* = 301)	Non-CVD (*n* = 132)	CVD (*n* = 169)	*P-*value
Age		54.0 [45.0, 64.0]	53.0 [44.0, 63.0]	55.0 [45.0, 65.0]	0.210
Sex					0.597
	Female	134 (44.5%)	56 (42.4%)	78 (46.2%)	
	Male	167 (55.5%)	76 (57.6%)	91 (53.8%)	
DM		83 (27.6%)	29 (22.0%)	54 (32.0%)	0.073
Hypertension		236 (78.4%)	104 (78.8%)	132 (78.1%)	0.999
Smoking		74 (24.6%)	34 (25.8%)	40 (23.7%)	0.777
Alcohol consumption		36 (12.0%)	17 (12.9%)	19 (11.2%)	0.799
SBP (mmHg)		142.0 [130.0, 157.0]	140.0 [129.0, 155.5]	145.0 [130.0, 160.0]	0.281
DBP (mmHg)		85.0 [77.0, 94.0]	88.0 [81.0, 96.6]	83.0 [74.0, 93.0]	0.001
BMI (kg/m^2^)		23.3 [20.4, 25.7]	23.2 [20.5, 25.4]	23.4 [20.3, 25.9]	0.730
Beta-receptor blockers		110 (36.5%)	61 (46.2%)	49 (29.0%)	0.003
Antiplatelet		68 (22.7%)	38 (28.8%)	30 (17.9%)	0.033
RAS blockers		73 (24.4%)	41 (31.3%)	32 (19.0%)	0.021
Lipid-lowering		87 (29.0%)	51 (38.6%)	36 (21.4%)	0.002
Hb (g/L)		89.0 [77.0, 104.0]	93.0 [81.0, 107.5]	87.0 [74.0, 102.0]	0.004
PLT (10^9^/L)		192.0 [142.0, 236.0]	191.5 [160.0, 234.0]	192.0 [133.0, 240.0]	0.418
ALB (g/L)		35.0 [31.5, 38.6]	35.3 [31.5, 39.3]	34.7 [31.8, 38.0]	0.349
BUN (μmol/L)		18.3 [12.3, 25.8]	17.2 [12.3, 23.7]	19.2 [12.3, 27.2]	0.152
Scr (μmol/L)		431.9 [285.3, 677.2]	389.1 [253.3, 570.2]	483.7 [301.7, 763.4]	0.006
UA (μmol/L)		434.2 [350.3, 527.4]	437.5 [348.3, 512.5]	429.6 [353.2, 529.1]	0.951
TC (mmol/L)		4.2 [3.5, 5.2]	4.4 [3.5, 5.3]	4.2 [3.5, 5.1]	0.156
TG (mmol/L)		1.4 [0.9, 2.1]	1.5 [0.9, 2.3]	1.4 [0.9, 1.9]	0.395
LDL (mmol/L)		2.4 [1.8, 3.1]	2.6 [1.9, 3.393]	2.29 [1.8, 2.9]	0.006
Calcium (mmol/L)		2.1 [1.9, 2.2]	2.1 [1.9, 2.2]	2.1 [1.9, 2.2]	0.656
Phosphorus (mmol/L)		1.5 [1.2, 1.8]	1.4 [1.2, 1.7]	1.5 [1.2, 1.8]	0.496
eGFR (ml/min)		13.8 [7.2, 24.6]	12.1 [8.4, 20.5]	15.8 [6.5, 26.0]	0.503
24 h urine protein (mg)		2,175.0 [1,062.0, 4,085.0]	2,305.0 [1,033.5, 4,076.8]	2,099.0 [1,100.0, 4,146.0]	0.839
PTH (ng/L)		231.8 [124.3, 411.4]	231.8 [108.8, 330.0]	231.88 [136.1, 486.0]	0.060
CRP (mg/L)		3.3 [2.9, 7.2]	3.1 [2.9, 5.3]	3.5 [3.1, 8.4]	<0.001
IVS (cm)		1.0 [1.0, 1.2]	1.0 [0.9, 1.1]	1.10 [1.0, 1.2]	<0.001

**Note:**

Abbreviations: CVD, cardiovascular disease; CKD, chronic kidney disease; DM, diabetes mellitus; SBP, systolic blood pressure; DBP, diastolic blood pressure; BMI, body mass index; RAS, renin-angiotensin aldosterone system; Hb, hemoglobin; PLT, platelet; ALB, albumin; BUN, blood urea nitrogen; Scr, serum creatinine; UA, uric acid; TC, total cholesterol; TG, triglycerides; eGFR, the estimated glomerular filtration rate; PTH, parathyroid hormone; CRP, C reactive protein; IVS, interventricular septal.

### Lasso regression of CVD events in patients with CKD stages 3–5 patients

**Figure 2:**
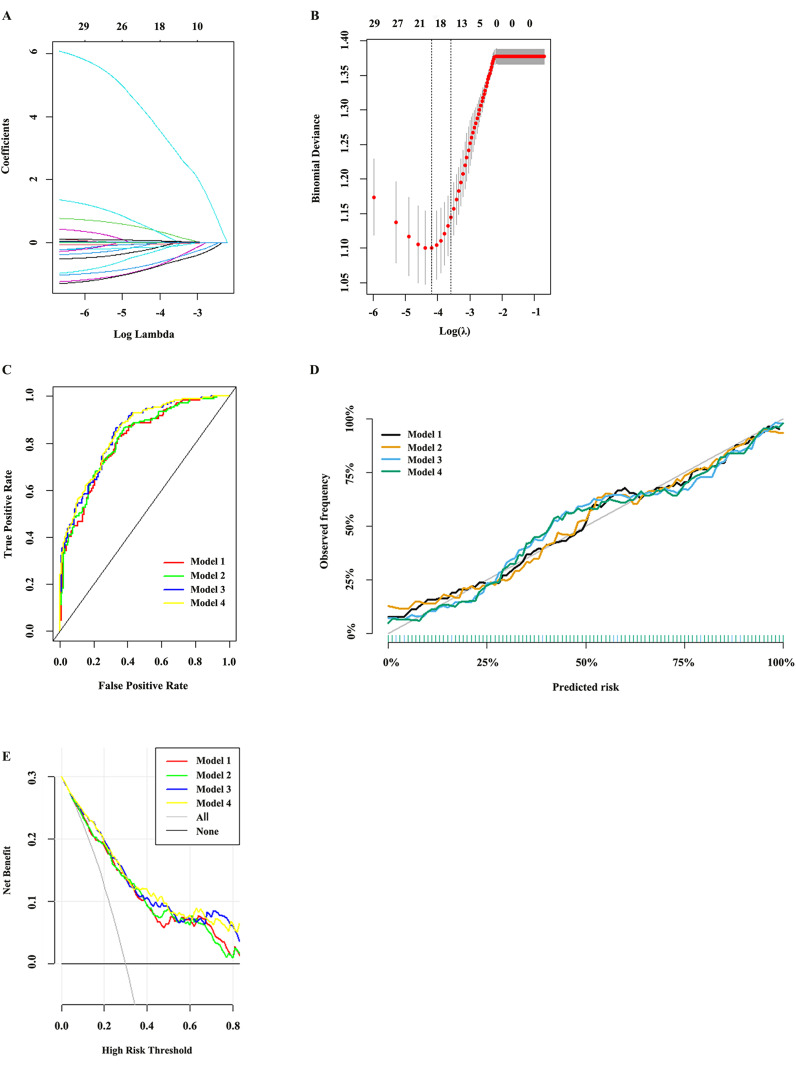
(A) LASSO regression coefficient distribution plot; (B) cross validation plot for LASSO regression; (C) the ROC curves for the four models; (D) the calibration curves for the four models; (E) the DCA curves for the four models.

This study defined whether CVD events occurred within 5 years in patients with CKD stages 3–5 as dependent variable. Age, gender, BMI, the history of diabetes mellitus, the history of hypertension, smoking, alcohol consumption, SBP, DBP and usage of β receptor blockers, antiplatelet agents, RAAS blockers, lipid-lowering agents, the levels of Hb, PLT, ALB, BUN, Scr, UA, total TC, TG, LDL-C, serum calcium, phosphorus, eGFR, 24 h urinary protein and PTH, CRP and cardiac IVS were defined the independent variables. To reduce the problem of collinearity of the independent variables, the variables were screened by lasso regression analysis in R 4.1.3. It indicated that gender, presence or absence of DM, history of smoking, SBP and DBP, and use or not β blockers, the use of antiplatelet drugs, the use of RAAS blockers, the use of lipid-lowering drugs, the levels of Hb, Scr, serum calcium, eGFR, PTH and C-reactive protein, the thickness of the cardiac interventricular septum are risk factors for incident CVD within 5 years in patients with CKD stages 3–5 (Figs. 2A and 2B).

### Multivariable logistic regression of CVD events in patients with CKD stages 3–5 patients

The 16 variables selected as statistically significant by the lasso regression analysis were further included in the multivariable logistic regression analysis and it indicated the history of DM, SBP and DBP, and use or not β blockers, the use of RAAS blockers, the use of lipid-lowering drugs, the levels of Scr, eGFR, PTH and C-reactive protein, the thickness of the cardiac interventricular septum were risk factors for incident CVD events within 5 years in patients with CKD stages 3–5 (*P* < 0.05); However, gender, smoking history, whether antiplatelet agents were used, absolute hemoglobin value, and serum calcium were not statistically significant in multivariable logistic regression (*P* > 0.05) (Table 2).

**Table 2:** The results of multivariate logistic regression model among patient s between CVD and non-CVD group.

Variables	OR	95% CI		*P*
		Lower	Upper	
Gender	0.677	0.330	1.371	0.281
DM	2.260	1.074	4.905	0.035
Smoking	0.490	0.212	1.106	0.089
SBP (mmHg)	1.033	1.014	1.054	0.001
DBP (mmHg)	0.922	0.890	0.951	<0.001
Beta-receptor blockers	0.346	0.178	0.654	0.001
Antiplatelet	0.784	0.372	1.648	0.520
RAAS blockers	0.229	0.097	0.513	<0.001
Lipid-lowering	0.320	0.144	0.687	0.004
Hb (g/L)	0.995	0.977	1.013	0.600
Scr (μmol/L)	1.003	1.001	1.004	<0.001
Calcium (mmol/L)	3.114	0.952	11.442	0.074
1/eGFR (per 0.01 unit increase)	1.117	1.076	1.164	<0.001
PTH (ng/L)	1.002	1.001	1.004	0.009
CRP (mg/L)	1.018	1.001	1.037	0.045
IVS (m)	324.064	35.242	365.688	<0.001

### Development and validation of the clinical prediction models

This study used 11 indicators that were statistically significant based on multivariable logistic regression analysis as independent variables and “CVD events within 5 years in patients with CKD stages 3–5” as the dependent variable to construct a clinical risk prediction model. Based on the filtered variables, and combined with clinically relevant indicators, a total of four clinical risk prediction models were established, which were defined as the original model, inflammatory model, imaging model, and complete model, respectively, and sequentially referred to as Model 1, Model 2, Model 3, and Model 4. The original model incorporated the history of DM, SBP, DBP, and use or not β blockers, the use of RAAS blockers, the use of lipid-lowering drugs, the levels of Scr, eGFR, PTH, which were determined by reviewing the available literature and combining clinical conditions (Tangri et al., 2011; Grams et al., 2018; Weiss et al., 2015; Mahmood et al., 2014; Kannel, 1999). The inflammation model was based on the original model with the addition of CRP and the imaging model with the addition of IVS, whereas the full model contained all indicators.

Regarding discrimination, plotting of the ROC curve was employed and the area under the curve (AUC) was calculated (Fig. 2C). Compared with the original and inflammatory models, the imaging model and the full model performed better, with the highest C-statistic of 0.840 and 0.845, respectively. The AUC value of the imaging model was 0.840, with the 95% confidence interval (CI) of [0.796–0.883]. The AUC value of the full model was 0.845, with the 95% CI of [0.802–0.888] (Table 3). Regarding calibration, we plotted calibration curves (Fig. 2D), calculated AIC and BIC values for the four models, and performed H-L tests on the four models. AIC values and BIC values were lowest for the imaging model and the full model compared to the original and inflammatory models. The AIC values were 321.821 and 311.531, and the BIC values were 353.600 and 356.017, respectively. The *P*-values of the H-L test of the four models were all greater than 0.05, and the goodness of fit test of the four models all passed, indicating better calibration (Table 3). In addition, the present study performed a DCA curve analysis to evaluate the clinical practical utility of the four models (Fig. 2E). The results suggested that the clinical net benefit was significantly improved in all four models.

**Table 3:** Performances of the four models with different combinations of predictive variables.

Variables	Model 1	Model 2	Model 3	Model 4
AUC	0.809	0.813	0.840	0.845
95% CI	[0.761–0.857]	[0.766–0.861]	[0.796–0.883]	[0.802–0.888]
AIC	336.787	334.743	312.821	311.531
BIC	372.830	375.522	353.600	356.017
Hosmer-Lemeshow test *P*-value	0.473	0.587	0.067	0.332

**Note:**

Model 1, the original model; Model 2, the inflammation model; Model 3, the imaging model; Model 4, the full model.

In this study, we further calculated the calibration slope, calibration intercept, and calibration-in-the-large for four curves. Model 1 exhibited a calibration slope of 0.99 and a calibration intercept of 0.01, indicating good overall calibration with the curve closely aligning with the diagonal line. Model 2 had a slope of 0.9 and an intercept of 0.05, showing a slight deviation in the low-risk range, but overall good calibration. Model 3 presented a slope of 0.85 and an intercept of 0.13, with average calibration and deviations in the mid-low risk area. Model 4 demonstrated a slope of 0.98 in the low-risk range and 0.92 in the mid-to-high risk range, with an intercept of 0.01, indicating good overall calibration and a slight deviation in the mid-to-high risk range. Therefore, the Model 1 and Model 4 have a better calibration degree.

Ultimately, we performed a comprehensive evaluation combining the discrimination, calibration, and clinical utility of the model, and the complete model was selected as the optimal one. The complete model incorporated the history of DM, SBP, DBP, and use or not β blockers, the use of RAAS blockers, the use of lipid-lowering drugs, the levels of Scr, eGFR, PTH, CRP and IVS. According to the sample size of this study, tenfold cross validation was selected for internal validation of the complete model, mainly by evaluating two aspects of model accuracy and consistency. The results suggested that the complete model had an accuracy of 0.728, and the accuracy was good; The kappa value of the complete model in consistency was 0.448, and the consistency was fair, which indicated that the complete model was established successfully.

### A nomogram of the full model

**Figure 3:**
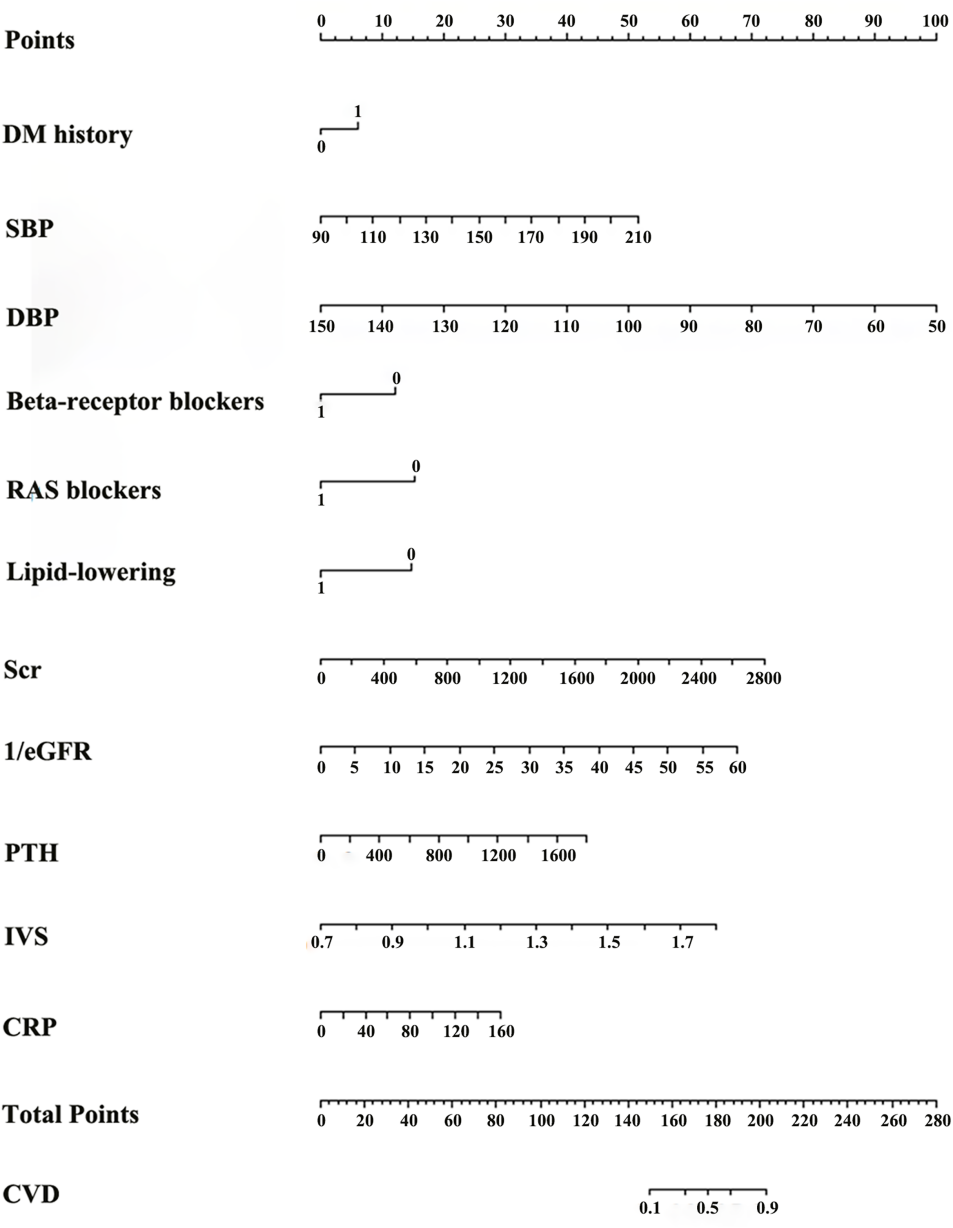
The nomogram for the full model.

Based on the above results, the full model exhibited better discrimination and calibration. Therefore, this study ranked the complete model as the optimal one. Based on the independent variables included in the full model, the history of DM, SBP and DBP, and use or not β blockers, the use of RAAS blockers, the use of lipid-lowering drugs, the levels of Scr, eGFR, PTH and C-reactive protein, the thickness of the cardiac interventricular septum were transformed according to functional relationships, after which the calibrated segments were used to construct nomograms in the plane according to the corresponding proportions in order for this risk prediction model to be better used in clinical practice. Using nomograms, we can visually and simply calculate the risk of CVD events over 5 years in patients with CKD stages 3–5 (Fig. 3). In our nomogram, each of the 11 patient-specific indicators is allocated a corresponding score as delineated in the ‘Points’ column. The aggregate of these individual scores yields a total point tally, which is then referenced against the ‘Total Points’ scale within the nomogram. Utilizing this cumulative score, the risk of cardiovascular events (CVD) for patients with chronic kidney disease (CKD) stages 3–5 is ascertained by aligning it with the ‘CVD’ probability column on the nomogram.

## Discussion

CKD is an increasingly prevalent chronic disease worldwide, affecting 15–20% of adults (Pugh, Gallacher & Dhaun, 2019). CKD is a strong, independent risk factor for CVD (Reiss et al., 2018). Patients with advanced CKD (stages 3–5) face a disproportionately high burden of CVD, driven by a complex interplay of traditional and CKD-specific pathophysiological mechanisms, such as mineral bone disorders, chronic inflammation, and uremic toxicity (Speer et al., 2022; Ridker et al., 2017; Matsushita et al., 2022). This context underscores the need for risk prediction tools specifically tailored to this population.

This study developed and internally validated a clinical risk prediction model for incident CVD events over a 5-year period in patients with CKD stages 3–5. It is specifically for patients with CKD stages 3–5 and this risk prediction model includes not only traditional risk factors influencing CVD events but also non-traditional risk factors associated with CKD specific complications. If validated externally, this model could potentially aid in the clinical screening of individuals at high risk of future CVD and inform consideration of earlier or more intensive management strategies, which might help delay CVD events, with the potential to reduce personal and societal economic burden. Importantly, our model integrates routine clinical variables with CKD-relevant markers (CRP, PTH) and an imaging parameter (IVS), providing a clinically deployable tool tailored to CKD biology. This approach complements specialized omics-based strategies by offering a practical instrument for broad implementation in routine care.

The predictors retained in our final model collectively reflect the multifactorial nature of CVD risk in advanced CKD. The strong predictive value of a history of diabetes is consistent with its established role as the most prominent cause of decreased kidney function and an independent risk factor for incident CVD (Fox et al., 2004; Nakamura et al., 2006; Weiner et al., 2004; Hahr & Molitch, 2022). Furthermore, CKD with DM is a stronger determinant of CVD than CKD without DM (Fox et al., 2012; Huang et al., 2021). Similarly, the significance of higher systolic blood pressure aligns with its long-recognized status as a key risk factor (László, Reusz & Nemcsik, 2016). Notably, the predictive signal for lower diastolic blood pressure may be indicative of increased arterial stiffness—a hallmark of vascular pathology in CKD that carries strong prognostic value and can manifest as a widened pulse pressure (Reiss et al., 2018; László, Reusz & Nemcsik, 2016; Düsing et al., 2021; Mathew et al., 2017).

The pathophysiology of atherosclerosis in CKD differs fundamentally from classical forms. Innate immune activation, chronic oxidative stress, uremic toxicity, and adverse vascular remodeling collectively accelerate plaque formation and instability. These mechanisms justify our inclusion of inflammatory (CRP), mineral metabolism (PTH), and cardiac structural (IVS) markers alongside traditional risk factors, as they capture key aspects of CKD-specific vascular injury. Moreover, the composite CVD endpoint used in this study reflects the diverse clinical manifestations of this complex pathophysiology.

The finding that not using guideline-recommended medications (β-blockers, RAAS inhibitors, and lipid-lowering drugs) was associated with higher risk is noteworthy. RAAS blockers are first-line for blood pressure control in CKD and may improve kidney outcomes (Gregg & Hedayati, 2018). β-blockers are preferred in conditions like sympathetic overactivity and heart failure (Ptinopoulou, Pikilidou & Lasaridis, 2013; Campbell, 2002; Tonelli et al., 2012). And lipid-lowering therapy significantly reduces CVD events in CKD (Shlipak et al., 2021; Baigent et al., 2011). Therefore, the absence of these therapies in our model likely identifies a higher-risk subgroup due to contraindications, intolerance, or gaps in care, rather than suggesting a lack of drug benefit. However, due to the observational nature of our study, these protective associations should be interpreted with caution, as they may be subject to confounding by indication—where patients receiving these medications may differ systematically from non-recipients in ways not fully captured by our covariates. Residual confounding cannot be ruled out, and these findings warrant confirmation in prospective studies.

Furthermore, our model directly incorporates markers of CKD severity and its specific complications: lower eGFR and higher serum creatinine anchor the risk to the degree of kidney function loss (Matsushita et al., 2010; Astor et al., 2011). The inclusion of elevated PTH captures the contribution of CKD-mineral bone disorder; elevated PTH levels are independently associated with CVD events in CKD (Kamycheva, Sundsfjord & Jorde, 2004; Rostand & Drüeke, 1999; Bhuriya et al., 2009). Similarly, elevated CRP captures the contribution of the chronic inflammation that is a landmark of CKD and a risk factor for its progression and CVD morbidity/mortality (Kannel, 1999; Reiss et al., 2018; Kamycheva, Sundsfjord & Jorde, 2004; Rostand & Drüeke, 1999; Bhuriya et al., 2009; Gupta et al., 2012; Akchurin & Kaskel, 2015). From a mechanistic perspective, dynamic models of atherosclerosis progression, such as the logistic intima-media thickness (IMT) growth model, demonstrate that inflammation serves as a key driver of the accelerated (inflection) phase of plaque growth, providing a framework to understand how elevated CRP levels may translate into heightened cardiovascular risk in the CKD setting (Formanowicz & Krawczyk, 2020). Finally, greater IVS thickness, a marker of left ventricular hypertrophy common in CKD, provides a direct link to cardiac end-organ damage. This structural remodeling can be conceptually linked to the accelerated phase of the IMT logistic growth curve described in dynamic models of atherosclerosis progression, as progressive intimal thickening increases arterial stiffness and afterload, ultimately contributing to left ventricular hypertrophy as captured by increased IVS thickness (Formanowicz et al., 2021).

It is important to position our model within the landscape of existing prediction tools. Unlike general population risk scores, such as Framingham, or models predicting kidney failure, our tool is designed specifically for 5-year CVD risk in CKD stages 3–5. While models like that from Grams et al. (2018) predict non-fatal CVD in CKD (15), our model’s integration of an imaging parameter (IVS) and a systemic inflammation marker (CRP) alongside routine clinical data offers a holistic assessment tailored to the advanced CKD phenotype. However, given that over half of our cohort (52.2%) had stage 5 CKD, the model’s performance may differ in earlier stages, and external validation in more diverse populations is warranted. Recent advances in proteomics and metabolomics have identified novel signatures that improve risk discrimination in CKD and map to immune, fibrotic, and metabolic pathways. While these omics approaches offer deep biological insights, their complexity and cost limit widespread adoption. Our model, built on routinely available variables, complements these advances by providing a practical and accessible tool for cardiovascular risk stratification in everyday clinical practice.

Importantly, our study goes beyond simple combination of known predictors by systematically quantifying the incremental predictive value of non-traditional CKD-specific factors through nested model comparisons. As shown in Table 3, the addition of CRP (Model 2) and IVS (Model 3) to the original model (Model 1) progressively improved discrimination, with the full model (Model 4) achieving the highest AUC (0.845) and lowest AIC (311.531). This stepwise improvement demonstrates that inflammation and subclinical cardiac remodeling are not merely epiphenomena but carry independent prognostic information beyond traditional risk factors and routine laboratory parameters. To our knowledge, this is the first risk prediction tool for CVD in CKD stages 3–5 that simultaneously incorporates clinical, inflammatory, and echocardiographic dimensions within a single model, and that empirically validates their additive contribution. By doing so, our model addresses a critical gap in existing literature, where prior tools have either omitted these domains or failed to demonstrate their incremental utility. This multidimensional approach aligns with the complex pathophysiology of CVD in advanced CKD and may enable more precise risk stratification, guiding individualized preventive strategies.

All in all, the occurrence of CVD events in CKD stage 3–5 patients is the result of the combined influence of multiple factors, and this study established and validated clinical risk prediction models by collecting data from patients in our hospital. Both traditional and non-traditional risk factors affecting CVD events were included in the model, and finally the model was transformed into a visualized nomogram, which was intended to be more intuitive and practical and could be beneficial for optimizing clinical management of CKD patients pending further validation.

Over the past decade, CKD–CVD risk research has evolved from traditional Framingham-based frameworks toward incorporating CKD-specific pathophysiological mechanisms, including mineral bone disorders, chronic inflammation, and uremic toxicity. Concurrently, advances in proteomics and metabolomics have uncovered novel molecular signatures that refine risk prediction and illuminate underlying biological pathways. Our study bridges these complementary perspectives by developing a multidimensional risk model that integrates routine clinical variables with CKD-relevant markers (CRP, PTH) and an imaging parameter (IVS). This approach provides a clinically deployable tool that captures key aspects of CKD-specific vascular injury while remaining accessible for broad implementation. As the field continues to move toward precision medicine, such practical instruments will be essential for translating biological insights into improved risk stratification and patient care. Future efforts should focus on external validation and exploring whether integration with emerging biomarkers could further enhance predictive performance.

While our study offers potential benefits by identifying high-risk individuals, several limitations must be acknowledged. First, the primary methodological limitation of this study lies in the use of logistic regression. This model predicts outcomes at a fixed time point (5 years) and cannot adequately account for differences in individual follow-up times or censored data, which may introduce bias. The choice of this model was based on the simplicity of its clinical interpretation and the aim of developing a fixed-time-point assessment tool. We acknowledge that survival analysis methods would better accommodate variable follow-up durations and censoring; however, we prioritized clinical interpretability and ease of implementation for a practical 5-year risk stratification tool. Consequently, the resulting risk estimates should be regarded as standardized estimates within the 5-year period. Second, the single-center, retrospective design with a cohort of 301 patients may limit generalizability and introduces the possibility of selection bias. External validation in independent populations is needed before clinical implementation. Third, and importantly, this study represents a model development and validation exercise. We have not tested the actual clinical impact or patient outcomes resulting from interventions guided by this model. Its ultimate clinical utility must be established through prospective studies evaluating whether its use leads to improved management decisions and better patient prognosis.

Fourth, we did not formally account for the competing risk of non-CVD death, which may influence absolute risk estimates. However, this does not alter the validity of our fixed-timepoint approach, as our primary aim was risk stratification rather than absolute risk prediction. Fifth, the predominance of stage 5 CKD (52.2%) in our cohort may limit the model’s transportability to earlier stages. Sixth, oxidative stress biomarkers (*e.g*., AOPPs, AGEs) were not assessed despite their established role in CKD-associated vascular injury; future studies should consider incorporating these markers. Seventh, as discussed in the medication section, the observed protective associations for β-blockers, RAAS inhibitors, and lipid-lowering drugs should be interpreted with caution due to potential confounding by indication. Finally, although we included both non-dialysis and dialysis patients to reflect real-world practice, we did not adjust for dialysis status in the model, which may introduce residual confounding. However, the similar proportion of dialysis patients between the outcome groups suggests that this may not have substantially biased the findings.

## Conclusions

This study established and validated a clinical risk prediction model based on variables readily available in clinical practice and aimed to predict the risk of CVD events over a 5-year period in patients with CKD stages 3–5. This practical and convenient tool combines traditional and non-traditional risk factors influencing CVD events, including the history of DM, SBP and DBP, and use or not β blockers, the use of RAAS blockers, the use of lipid-lowering drugs, the levels of Scr, eGFR, PTH and C-reactive protein, the thickness of the cardiac interventricular septum. This risk prediction model is helpful for early identification of patients at high risk of CKD and for prevention and delay of progression and occurrence of CVD events in CKD patients, thereby improving the outcomes of CKD patients.

### Declarations

This study involves the development and validation of a prediction model. Its design, conduct, analysis, and reporting adhere to the requirements of the Transparent Reporting of a multivariable prediction model for Individual Prognosis Or Diagnosis (TRIPOD) statement. The completed TRIPOD checklist is provided as Supplemental Material.

## Supplemental Information

10.7717/peerj.21312/supp-1 Supplemental Information 1 CKD-CVD RAW DATA.

10.7717/peerj.21312/supp-2 Supplemental Information 2 Categorical data.

10.7717/peerj.21312/supp-3 Supplemental Information 3 R-code.

10.7717/peerj.21312/supp-4 Supplemental Information 4 STROBE checklist.

10.7717/peerj.21312/supp-5 Supplemental Information 5 TRIPODAI checklist.

## Additional Information and Declarations

### Data Availability

The following information was supplied regarding data availability:

The raw measurements are available in the Supplemental File.
