# Supplementary material for: A retrospective study on development and internal validation of cardiovascular disease risk prediction model for patients with chronic kidney disease stage 3–5 within 5 years
Source: PeerJ. 2026 Jun 5;14:e21312. doi: 10.7717/peerj.21312 (PMC13245425; doi:10.7717/peerj.21312)
Supplement: Supplemental Information 2 [file peerj-14-21312-s002.doc]

Variable: outcome

Codes:

0 = non-CVD

1 = CVD

Variable: sex

Codes:

0 = male

1 = female

Variable: TNB

Codes:

0 = no diabetes

1 = diabetes

Variable: GXY

Codes:

0 = no hypertension

1 = hypertension

Variable: XYS

Codes:

0 = no smoking

1 = smoking

Variable: YJS

Codes:

0 = no drinking

1 = drinking

Variable: X1

Codes:

0 = no Beta-receptor blockers

1 = Beta-receptor blockers

Variable: X2

Codes:

0 = no Antiplatelet

1 = Antiplatelet

Variable: X3

Codes:

0 = no RAS blockers

1 = RAS blockers

Variable: X4 Codes:

0 = no Lipid-lowering

1 = Lipid-lowering
